# Heavy Ions Induced Single-Event Transient in SiGe-on-SOI HBT by TCAD Simulation

**DOI:** 10.3390/mi16050532

**Published:** 2025-04-29

**Authors:** Yuedecai Long, Abuduwayiti Aierken, Xuefei Liu, Mingqiang Liu, Changsong Gao, Gang Wang, Degui Wang, Sandip Majumdar, Yundong Xuan, Mengxin Liu, Jinshun Bi

**Affiliations:** 1 School of Integrated Circuits, Guizhou Normal University, Guiyang 550025, China; 232200071392@gznu.edu.cn (Y.L.); erkin@gznu.edu.cn (A.A.); 201307129@gznu.edu.cn (X.L.); mq.liu@gznu.edu.cn (M.L.); gaochangsong@gznu.edu.cn (C.G.); 202107021@gznu.edu.cn (G.W.); 201707003@gznu.edu.cn (D.W.); 2 Department of Physics, Serampore Girls’ College, Serampore 712201, India; sandipiitkgp13@gmail.com; 3 Institute of Microelectronics, Chinese Academy of Sciences, Beijing 100029, China; 4 College of Integrated Circuits, University of Chinese Academy of Sciences, Beijing 100049, China; 5 Beijing Zhongke New Micro Technology Development Co., Ltd., Beijing 100029, China

**Keywords:** SiGe heterojunction bipolar transistor, silicon-on-insulator, single-event transient, heavy ions

## Abstract

In this work, the effects of heavy ion strike position, incident angle, linear energy transfer (LET) value, ambient temperature, bias conditions, and the synergistic effects of total dose irradiation on the single-event transient (SET) in silicon-germanium heterojunction bipolar transistors on silicon-on-insulator (SiGe-on-SOI HBTs) were investigated using TCAD simulations. It was demonstrated that, compared to the bulk SiGe HBT, the SiGe-on-SOI HBT exhibits lower transient current and less charge collection, indicating better resistance to SET. The SET response is more pronounced when heavy ions strike vertically from the emitter and base regions. Transient current and collected charge escalate with increasing incident angle, demonstrating a strong linear correlation with LET values. As the temperature decreases, the peak transient current increases, while the pulse duration decreases and the total collected charge diminishes. After total dose irradiation, the peak transient current in the SiGe-on-SOI HBT decreases, whereas the damage was more severe in the absence of irradiation. Under collector positive bias and positive bias, significant SET responses were observed, while cutoff bias and substrate bias exhibited better resistance to SET damage. These findings provide critical insights into radiation-hardened design strategies for the SiGe-on-SOI HBT.

## 1. Introduction

The SiGe HBT is a critical component for aerospace applications and has been recognized as a highly promising candidate for the development of electronic systems designed for extreme environments. These characteristics include low noise, high speed, low cost, and high integration density and demonstrate exceptional operational stability across an extensive temperature range, particularly under extreme environmental conditions [1].

SiGe HBTs exhibit remarkable tolerance to both enhanced low-dose-rate-sensitivity (ELDRS) effects and displacement damage, demonstrating superior radiation hardness compared to conventional silicon-based devices [2,3,4,5], but in SiGe HBT digital circuits, the devices demonstrate significant susceptibility to single-event upsets (SEUs) and SET [6]. These limitations significantly hinder their application and development in space environments. SiGe-on-SOI HBTs are attractive for mixed-signal radio frequency (RF) applications [7] and have garnered significant research attention due to their compatibility with SOI CMOS technology [8]. SiGe-on-SOI HBT technology has emerged as a research focus for extreme environment applications.

There are some published papers on SET characteristics between bulk and thick-film SiGe-on-SOI HBT technologies from John D. Cressler’s group. Experimental results demonstrated that thick-film SiGe-on-SOI HBTs achieved an approximately 350-fold reduction in sensitive volume compared to bulk silicon counterparts [9]. They first reported the high-energy proton (63 MeV) irradiation response of thin-film SiGe-on-SOI HBTs, revealing *β* degradation caused by an increased base current with proton fluence. Radiation-induced net positive charge in the buried oxide (BOX) layer altered local electron density and electric field distribution, thereby delaying the Kirk effect. Notably, proton irradiation enhanced both *f*_T_ and *f*_max_ in SiGe-on-SOI HBT technology [10]. Using Texas Instruments’ complementary SiGe-on-SOI technology with inverse-mode PNP HBTs, significant improvement in single-event transient tolerance was achieved in current mirror circuits [11]. For high-voltage (>30 V) SiGe-on-SOI platforms, the BOX layer eliminates the large-area collector-substrate junction, enabling reliable operation above 200 °C. At 300 °C, reduced current-induced damage and annealing recovery characteristics were observed [12]. Furthermore, combined high-temperature operation and self-heating effects showed no significant degradation in SET tolerance for SiGe-on-SOI HBT technology [13].

The removal of the collector-substrate junction due to BOX in SiGe-on-SOI HBTs effectively reduces the susceptibility of a bulk SiGe HBT to SEE [14]. However, studies have revealed that SiGe-on-SOI HBTs exhibit distinct charge collection mechanisms of single-event effects compared to their bulk silicon counterparts [15]. Significant research gaps persist in understanding the SEE radiation response of SiGe-on-SOI HBT technology. It can be inferred that the sensitivity factors and their coupling mechanisms with extreme temperature conditions in SiGe-on-SOI HBTs will manifest radiation response characteristics fundamentally divergent from those of conventional SiGe HBT architectures. In this paper, the effects of heavy ion strike position, incident angle, linear energy transfer (LET) value, ambient temperature, TID synergistic effects, and bias state on the SET in SiGe-on-SOI HBTs were investigated using Technology Computer Aided Design (TCAD) simulations.

## 2. Device Structure Model and Numerical Simulation of SET

### 2.1. SiGe-on-SOI HBT Device Structure Model

**Figure 1:**
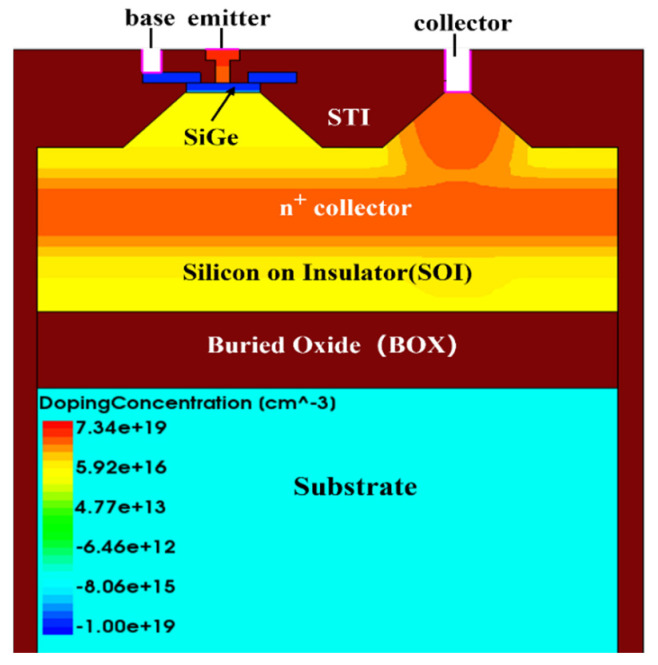
Schematic of the NPN SiGe-on-SOI HBT device structure.

This paper establishes a two-dimensional simulation model for NPN SiGe-on-SOI HBTs using Sentaurus TCAD. The structure of this model is the same as that of the traditional vertical SiGe HBT, as shown in Figure 1.

The base region is formed by a graded composition of Ge atoms, creating a SiGe base, where the Ge concentration increases gradually from 0% at the emitter/base (E/B) junction to 20% at the base/collector (B/C) junction. The boron doping concentration follows a Gaussian distribution, peaking at 2 × 10^19^ cm^−3^ in the middle and decreasing to a boundary concentration of 1 × 10^17^ cm^−3^, with a width of 90 nm. The polysilicon epitaxial base region has a width of 90 nm and a doping concentration of 1 × 10^19^ cm^−3^, serving as the base contact. Above the base region lies the polysilicon emitter, heavily doped with arsenic at a concentration of 6.34 × 10^19^ cm^−3^ to form the emitter contact. The doping concentration decreases following a Gaussian distribution to 1 × 10^19^ cm^−3^ at the edge of the base region, with a width of 300 nm. Below the base region is the larger collector region, with a width of 2 μm. The central part of the collector region features a Gaussian distribution of arsenic doping, with a peak concentration of 1 × 10^19^ cm^−3^ and an edge concentration of 1 × 10^16^ cm^−3^. The oxide layer has a thickness of 0.7 μm, and the substrate is uniformly doped with boron at a concentration of 6.68 × 10^16^ cm^−3^, with a width of 2 μm. Detailed NPN SiGe-on-SOI HBT parameters are listed in Table 1.

The selection of physical models plays a critical role in ensuring the accuracy of device simulation results. The physical models employed in TCAD simulations include the Philips unified mobility mode, and the Auger and Shockley–Read–Hall (SRH) recombination models [16]. Due to the presence of high carrier density gradients and germanium doping, which lead to gradual changes in the band structure, the velocity saturation model and bandgap narrowing model are also included [17].

**Figure 2:**
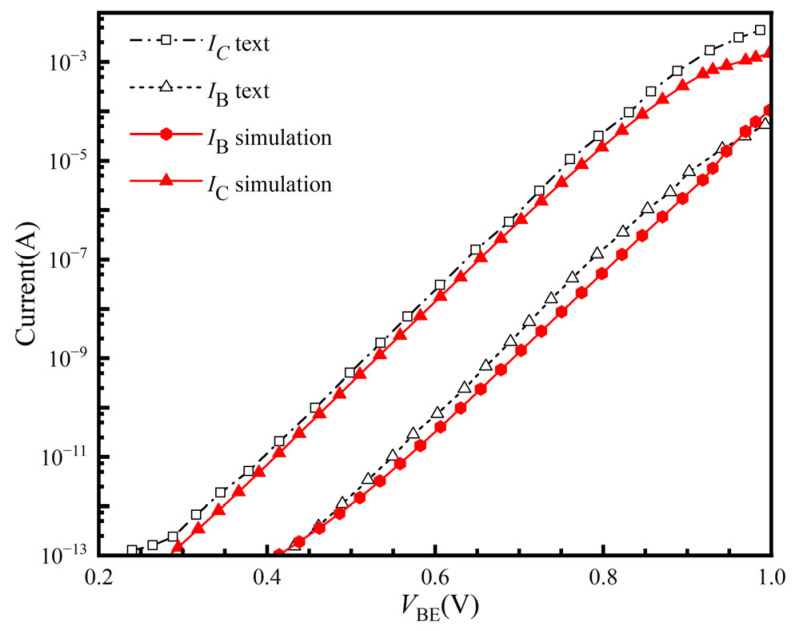
Input characteristics obtained by simulation and tests.

The prototype device used to build the simulation model is the IBM 8HP SiGe HBT. Figure 2 shows the Input characteristic curves obtained by simulation are calibrated by curves measured by KEITHLEY 4200 [18]. The simulated base and collector current exhibit good agreement with the actual experimental measurements within reasonable error margins. This validates the model’s suitability for subsequent radiation effects simulations.

### 2.2. Numerical Simulation of SET

When heavy ions strike a microelectronics device, they collide with semiconductor materials along their trajectory, generating electron–hole pairs. When the trajectory of the incident particle passes through or near the sensitive junction (pn junction) of the device, the electron–hole pairs generated by ionization along the trajectory are collected by the sensitive junction under the influence of the depletion region’s electric field. If the collected charge exceeds a certain critical value, a SEE is triggered [19]. In the TCAD tool, a physical module is included for simulating SEE induced by heavy ions. Based on the formula for the generation rate of electron–hole pairs when heavy ions strike semiconductor devices, the generation rate of electron–hole pairs accompanying heavy ion incidence can be determined [20], as shown in Equation (1).

(1) $$G\left(l,w,t\right)=G_{LET}\left(l\right)R\left(w,l\right)T\left(t\right)$$

In the equation, *l* represents the distance from a point on the ionization trajectory to the incident point, and *w* represents the radius of the ion track. *R*(*w*,*l*) and *T*(*t*) represent the spatial and temporal distribution functions of the carrier generation rate, respectively. *G_LET_*(*l*) is the LET generation rate, measured in pairs/cm^3^ [21]. By incorporating the PicoCoulomb instruction, the unit pairs/cm^3^ are converted to pC/μm. *R*(*w*,*l*) is defined as a Gaussian function, as shown in Equation (2).

(2) $$R\left(w,l\right)=\exp\left(-{\left(\frac{w}{w_{t}\left(l\right)}\right)}^{2}\right)$$

In Formula (2), *w* is the radial distance of the track, and *w_t_*(*l*) is the characteristic distance. As shown in Formula (3), *T*(*t*) is defined as the Gaussian function.

(3) $$T\left(t\right)=\frac{2\cdot \exp\left(-{\left(\frac{t-t_{0}}{\sqrt{2}\cdot s_{hi}}\right)}^{2}\right)}{\sqrt{2}\cdot s_{hi}\sqrt{\pi }\left(1+erf\left(\frac{t_{0}}{\sqrt{2}\cdot s_{hi}}\right)\right)}$$

In Equation (3), *t*_0_ represents the time at which the heavy ion begins to strike, and *s_h__i_* is the Gaussian characteristic value. LET denotes the linear energy transfer generation density. By integrating the aforementioned physical models and substituting the carrier generation rate calculated from Equation (1), the Poisson equation and continuity equation are solved to obtain the relationship between current and charge collection as a function of time.

## 3. Results and Discussion

### 3.1. Comparison Between Bulk SiGe HBT and SiGe-on-SOI HBT

Two-dimensional models of a SiGe HBT and SiGe-on-SOI HBT were established in TCAD. Compared to the bulk SiGe HBT, the SiGe-on-SOI HBT incorporates an additional BOX, while other structural parameters of the device remain the same. With all terminals of the device grounded, heavy ions were vertically incident from the emitter, penetrating through the bottom of the device. The charge deposition was set to 0.2 pC/μm, equivalent to a LET value of 20 MeV·cm^2^/mg.

**Figure 3:**
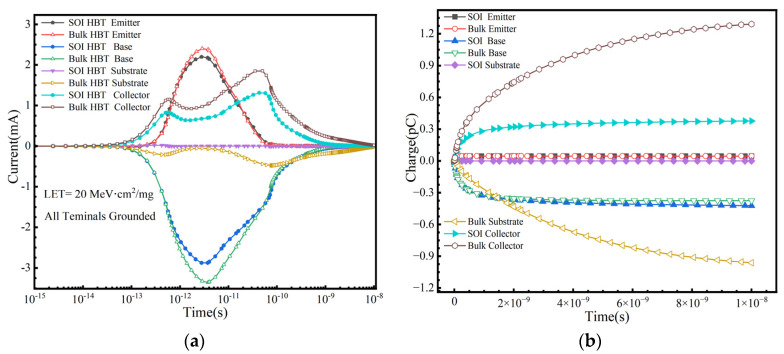
(**a**) The transient current and (**b**) the charge collection of heavy ions from the emitter center incident silicon SiGe HBT and SIGe-on-SOI HBT with respect to time.

From Figure 3a,b, the transient current and collected charge at each electrode over time can be observed after ion incidence. The peak transient currents at the collector, substrate, and base of the bulk SiGe HBT are greater than those of the SiGe-on-SOI HBT, while the difference at the emitter is relatively small. In the bulk SiGe HBT, the charge collected by the diffusion mechanism results in a smoother peak in the collector and substrate currents following the drift current peak. The collected charges at the collector and substrate are 1.29 pC and −0.96 pC, respectively. In contrast, for the SiGe-on-SOI HBT, the collector charge is 0.377 pC, representing a reduction of approximately four times, and the substrate charge is negligible. The charge collection in the bulk SiGe HBT is significantly higher than that in the SiGe-on-SOI HBT, primarily because the BOX layer eliminates the collector/substrate (C/S) junction [14], weakening the electric field in the collector region and thereby mitigating the SET effect. The charge collection at the base and emitter is similar for both devices. The results demonstrate, as expected, that the SiGe-on-SOI HBT effectively enhances resistance to SET.

### 3.2. The Influence of Heavy Ion Strike Positions

**Figure 4:**
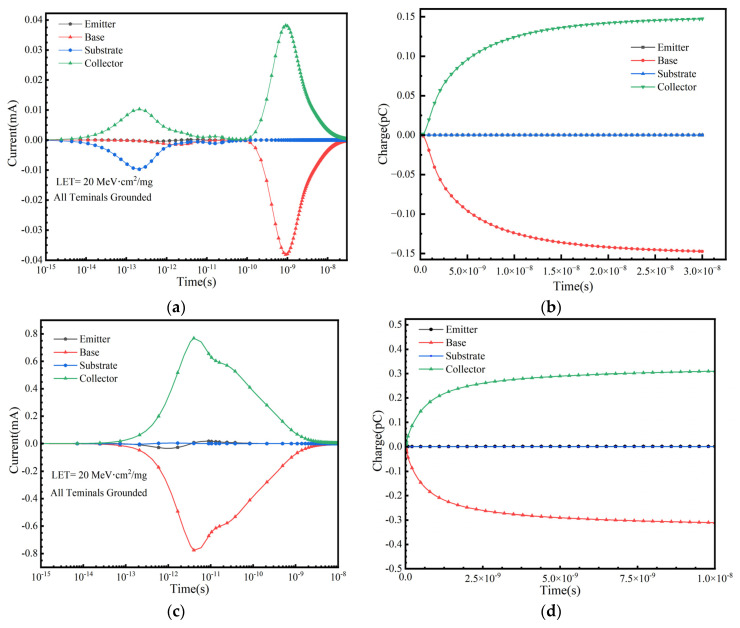
Transient current and collected charge as functions of time at each electrode are characterized by heavy ions vertically striking the central regions of (**a**,**b**) the collector and (**c**,**d**) the base.

The transient current and charge collection at each electrode of the SiGe-on-SOI HBT are closely related to the strike position of the heavy ions. By analyzing the simulation results of heavy ions striking at different positions, the sensitive regions of the SiGe HBT to SEE can be identified. With the heavy ion LET fixed at 20 MeV·cm^2^/mg and all terminals grounded, the transient current and collected charge over time were obtained for vertical strikes at the center of the base and collector regions. Unless otherwise stated, the same conditions apply in the following discussion. The results are shown in Figure 4. When heavy ions strike vertically at the center of the emitter in the SiGe-on-SOI HBT, electrons are collected by the emitter and collector, while holes are collected by the base and substrate. When heavy ions strike at the center of the collector and base, the transient current and collected charge at the substrate and emitter are minimal. Electrons are collected by the collector, and holes are collected by the base. When heavy ions strike the collector, two peak current pulses are observed due to the transient current formed by carrier drift and diffusion mechanisms. The smallest transient current peak and collected charge were observed when heavy ions struck the collector. The results indicate that the SiGe-on-SOI HBT is most sensitive to single-event transients when striking the emitter, followed by the base and collector injections. This is because a strike at the center of the emitter traverses all electrode regions, enabling rapid collection of electron–hole pairs through drift, which results in a larger transient current peak [3].

### 3.3. The Influence of Heavy Ion Incident Angles

**Figure 5:**
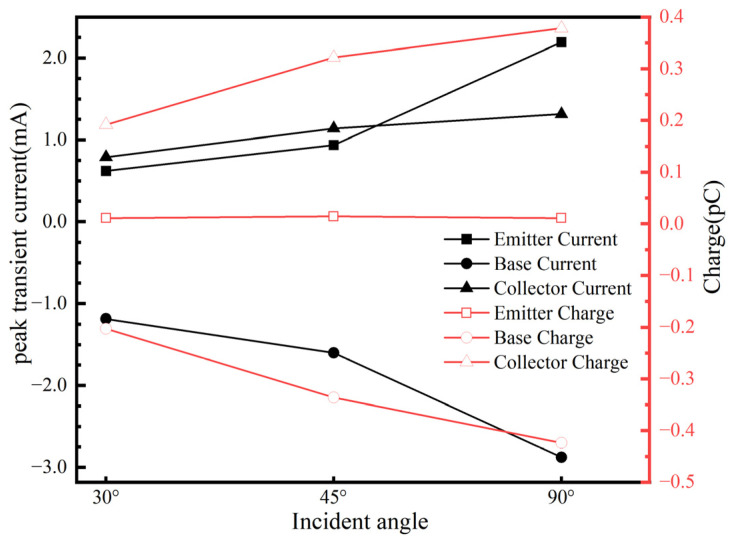
Changes in peak transient current of each electrode and collected charge with time at different incidence angles.

The impact of heavy ion incident angles on the sensitivity of the SiGe-on-SOI HBT to SEE is closely related [22]. To investigate the influence of different incident angles on SET, the horizontal direction is defined as 0°, with the incident angle increasing in a clockwise direction, and the vertical incidence as 90°. The heavy ion LET value is fixed at 20 MeV·cm^2^/mg, and the ions strike from the center of the emitter at incident angles of 30°, 45°, and 90°. Since the effective LET value of heavy ions striking the surface of the SiGe-on-SOI HBT is influenced by the incident angle, and the length of the ion track in the sensitive region of SiGe-on-SOI HBT varies with the incident angle, the peak transient current and collected charge at each electrode are affected. As shown in Figure 5, as the incident angle increases, the peak transient current and collected charge also increase. This is because vertical incidence simultaneously traverses the sensitive regions of the E/B and B/C junctions, leaving a longer ionization track in the sensitive volume, thereby generating more ionization energy deposition and excess carriers [23]. Consequently, SET exhibits maximum vulnerability under vertical incidence conditions.

### 3.4. The Influence of Heavy Ion LET Values

**Figure 6:**
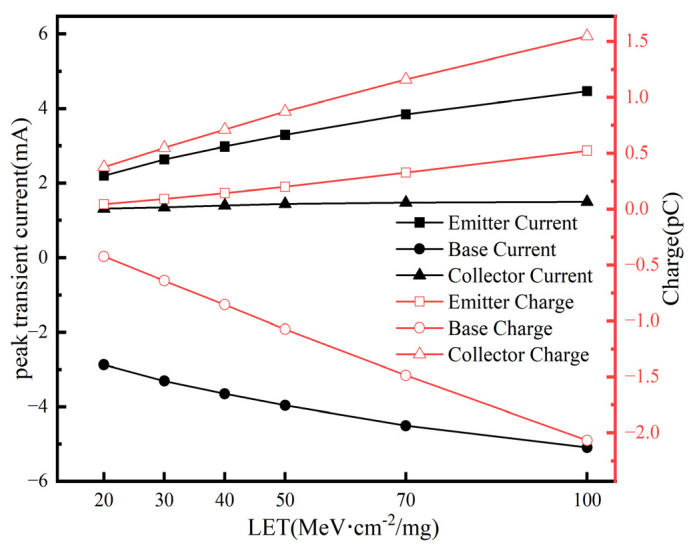
The peak transient current and collected charge of each electrode under different LET values.

The energy deposition of heavy ions is one of the critical factors influencing the sensitivity of SEE [24]. Therefore, the impact of heavy ions with different LET values on the SiGe-on-SOI HBT was investigated by vertically striking the center of the emitter in the sensitive region and observing the peak transient current and collected charge at each electrode. As depicted in Figure 6, the peak transient currents and collected charges of the electrodes exhibit a proportional increase with rising LET values, demonstrating a strong linear correlation with the LET magnitude. This is because the number of electron–hole pairs generated by heavy ions with different LET values in the SiGe-on-SOI HBT varies, and the quantity of electrons and holes increases with higher LET values. It can be inferred that the higher the LET of the heavy ions, the greater the energy deposition in the sensitive region of the emitter [25]. Consequently, more electron–hole pairs are generated in the sensitive region of the emitter. At an LET of 100 MeV·cm^2^/mg, the transient pulse current at the emitter is approximately 4.5 mA, and the charge collection time increases, with the collected charge reaching about 2 pC.

### 3.5. The Influence of Ambient Temperature

**Figure 7:**
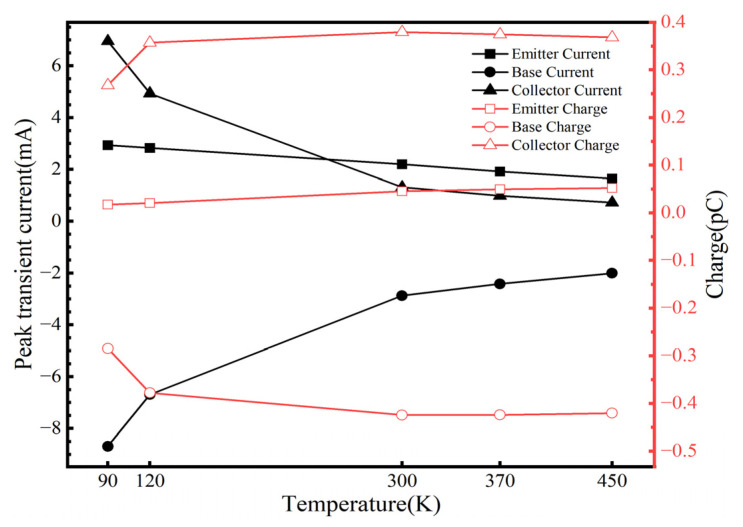
The peak transient current and collected charge of each electrode under different temperatures.

The carrier mobility in the SiGe-on-SOI HBT is influenced by temperature, making the ambient temperature during heavy ion irradiation a significant factor affecting the SET in the SiGe-on-SOI HBT. To study the impact of temperature on SET, heavy ions with a LET value of 20 MeV·cm^2^/mg were vertically incident at the center of the emitter, and the effects of temperatures, such as 90 K, 120 K, 300 K, 370 K, and 450 K, on SET were analyzed. As shown in Figure 7, the peak transient current at each electrode increases with decreasing temperature, while the pulse duration decreases, and the total collected charge diminishes. This phenomenon arises because, at lower temperatures, carrier mobility is minimally affected by scattering, resulting in a significant increase in drift velocity. The drift velocity of carriers is related to the time required for charge collection [26]. Consequently, enhanced carrier collection rates at the electrodes lead to higher transient currents and accelerated collection speeds [13].

### 3.6. Synergistic Effects of TID and SET

**Figure 8:**
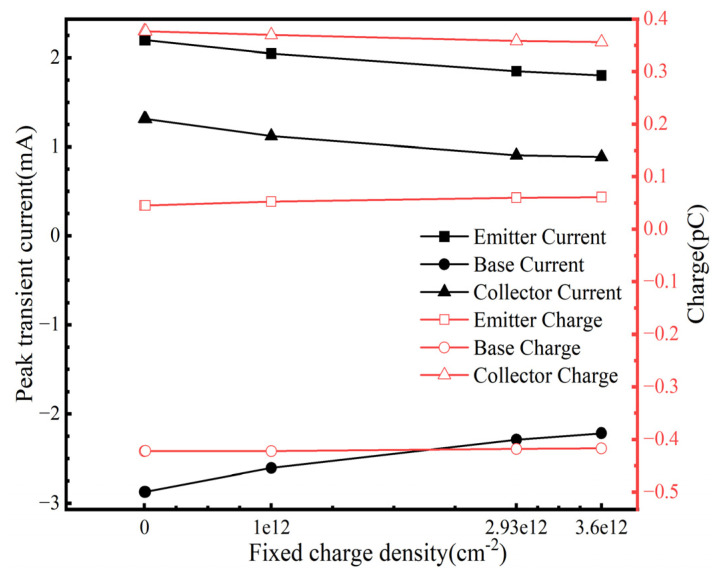
The peak transient current and collected charge as functions of fixed positive charge concentration.

When SiGe-on-SOI HBTs are used in extreme environments, they are exposed to mixed radiation fields, leading to complex synergistic effects. TID can introduce positive oxide charges and interface trap charges in the oxide layer [27], creating an additional electric field that influences SET. In the SiGe-on-SOI HBT, interface states play a dominant role. To study the impact of TID on SET, fixed positive charges were added at the Si/SiO_2_ interface and oxide layer [28], with concentrations of 1 × 10^12^ cm^−2^, 2.9 × 10^12^ cm^−2^, and 3.6 × 10^12^ cm^−2^ [29]. As shown in Figure 8, with the increase in fixed positive charge concentration, the peak transient current decreases, while the collected charge shows no significant difference. The space charge field induced by TID counteracts the funneling electric field, suppressing drift effects and attenuating transient current peaks. Interface traps reduce diffusion-mediated charge collection through recombination [30]. However, as the excess carrier density generated during SEE far exceeds the interface trap density, this effect is marginal, resulting in negligible differences in the collected charge.

### 3.7. The Response of SET Under Different Operating Bias Conditions

**Figure 9:**
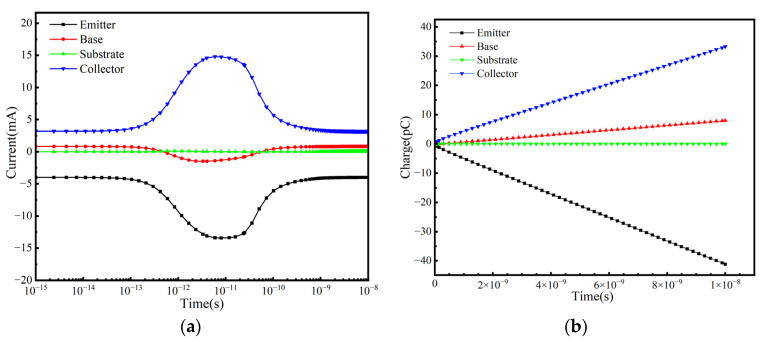
Positive bias: (**a**) Transient current at each terminal and (**b**) collected charge as a function of time.

**Figure 10:**
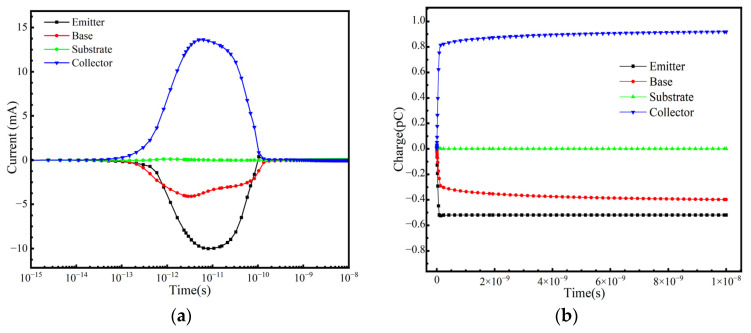
Collector positive bias: (**a**) Transient current at each terminal and (**b**) collected charge as a function of time.

**Figure 11:**
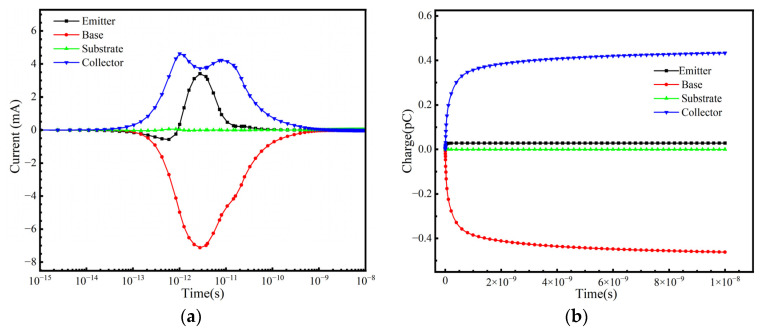
Cutoff bias: (**a**) Transient current at each terminal and (**b**) collected charge as a function of time.

**Figure 12:**
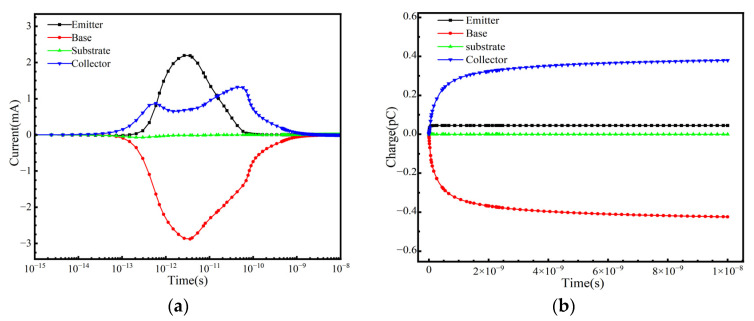
Substrate reverse bias: (**a**) Transient current at each terminal and (**b**) collected charge as a function of time.

In practical space applications, a SiGe-on-SOI HBT operates under various conditions, and the SET response is highly dependent on the bias conditions applied during irradiation. This study considers the SET response under the following bias conditions: positive bias (V_BE_ = +1.2 V, V_CE_ = +3 V, V_E_ = 0 V); cutoff bias (V_BE_ = −3 V, V_BC_ = −3 V); collector positive bias (V_CE_ = +3 V, V_E_ = 0 V, V_B_ = 0 V); and substrate reverse bias (V_S_ = −3 V). Figure 9, Figure 10, Figure 11 and Figure 12 illustrate the variation in terminal currents and collected charge over time under different bias conditions. The results show that under substrate reverse bias and cutoff bias, electrons are collected at the emitter and collector, while holes are collected at the base. Under collector positive bias, electrons are collected at the collector, and holes are collected at the emitter and base. Under positive bias, electrons are collected at the collector, while holes are collected at the base and emitter. These differences arise because when ions strike the sensitive region of the device, electron–hole pairs are generated. Electrons are collected by the positively biased regions, while holes flow toward lower potential regions. The internal electric field direction and carrier transport mechanisms vary under different bias conditions, leading to different SET responses [31].

For a systematic comparison, peak transient current increment (∆*I*_SET_ = *I*_total_ − *I*_static_) and collected charge are tabulated in Table 2 and Table 3. The transient current increment under positive bias is smaller than that under collector positive bias; however, the total collected charge is greater. This is because the strong electric field under collector positive bias accelerates the transient current peak, whereas the stable carrier transport path under positive bias results in higher charge collection efficiency. Under cutoff bias, the transient current increment is greater than that under substrate reverse bias. The charge collection at both the base and collector under cutoff bias is higher than that under substrate reverse bias, while the charge collection at the emitter is relatively small and shows no significant difference. The aforementioned results indicate that different bias states not only affect the transient current peaks and collected charge quantities at each terminal but are also related to the types of carriers collected at each terminal [17].

## 4. Conclusions

This paper analyzes the SET response of a SiGe-on-SOI HBT under various influencing factors by TCAD simulations. The results indicate that, compared to a bulk SiGe HBT, the SiGe-on-SOI HBT exhibits superior resistance to SEE. The most sensitive region for SEE is when heavy ions strike the emitter, and SET damage increases with the incident angle. Both the peak transient current and collected charge increase with higher LET values. At low temperatures, the transient current is higher, but the collected charge is lower than at high temperatures. After total dose irradiation, the peak transient current of SET is reduced. Under different bias conditions, positive bias and collector positive bias exhibit higher SET damage, followed by cutoff bias and substrate bias. These results provide guidance insights into the application and improvement in SiGe-on-SOI HBT devices for extreme space radiation environments.

## Figures and Tables

**Table 1:** SiGe-on-SOI HBT structure parameters.

Region	Thickness (μm)	Materials	Concentration (cm^−3^)
Substrate	2	Silicon	6.68 × 10^16^
BOX	0.7	Oxide	0
SiGe Base	0.09	SiGe	2 × 10^19^
Base	0.09	Poly silicon	1 × 10^19^
Collector	2	Silicon	1 × 10^16^
Emitter	30	Poly silicon	6.34 × 10^19^

**Table 2:** Peak transient current increment under different bias states.

Bias State	Emitter (mA)	Collector (mA)	Base (mA)
Positive bias	9.42	11.65	2.29
Collector positive bias	10.00	13.61	4.11
Cutoff bias	3.41	4.62	7.12
Substrate reverse bias	2.19	1.31	2.87

**Table 3:** Collected charge under different bias states.

Bias State	Emitter (pC)	Collector (pC)	Base (pC)
Positive bias	−1.157	1.408	−2.466
Collector positive bias	−0.519	0.918	−0.398
Cut off bias	0.028	0.433	−0.461
Substrate reverse bias	0.045	0.379	−0.424

## Data Availability

The data presented in this study are available on request from the corresponding author.
